# Shoseiryuto Promotes the Formation of a Tight-Junction Barrier in Cultured Human Bronchial Epithelial Cells

**DOI:** 10.1155/2023/4694243

**Published:** 2023-10-21

**Authors:** Jingya Lu, Ailing Hu, Takuji Yamaguchi, Masahiro Tabuchi, Yasushi Ikarashi, Hiroyuki Kobayashi

**Affiliations:** Department of Personalized Kampo Medicine, Juntendo University Graduate School of Medicine, Tokyo 113-8421, Japan

## Abstract

Shoseiryuto (SST) (Xiao-Qing-Long-Tang in Chinese) is an effective treatment for respiratory diseases, such as bronchial asthma and allergic rhinitis, but its effects on the bronchial tight-junction (TJ) barrier have not been clarified. This study aimed to evaluate the effect of SST on TJ-barrier function in human bronchial epithelial (16HBE) cells. The 16HBE cells were cultured in a culture medium without (control) and with SST in the absence and presence of bacterial endotoxin lipopolysaccharide (LPS) in transwell chambers. Transepithelial electrical resistance (TEER) and sodium fluorescein (Na-F) permeability of the cultured-cell monolayer were measured as TJ integrity markers. In addition, immunofluorescence staining and quantitative real-time polymerase chain reaction analysis were used to measure the expression of the TJ protein, occludin. SST increased TEER and decreased Na-F permeability of the 16HBE cell monolayers. Furthermore, SST increased both occludin mRNA and immunostained protein expressions, suggesting that SST has the effect of directly promoting epithelial TJ-barrier function. LPS decreased TEER, increased Na-F permeability, and decreased both occludin mRNA and protein expression. LPS-induced barrier dysfunction was completely blocked by pre/co- and posttreatment with SST. These results suggest that SST has protective and therapeutic effects against LPS-induced TJ-barrier damage. To our knowledge, these are the first results to demonstrate the protective and therapeutic effects conferred by TJ-barrier promoting, which may be a novel mechanism contributing to the efficacy of SST for respiratory diseases.

## 1. Introduction

Bronchial epithelial cells not only form respiratory pathways for oxygen to the alveoli but also a physical barrier separating the inside and outside of the body. This epithelial barrier helps prevent the invasion of various external factors, such as bacteria, viruses, allergens, and harmful substances [1].

For the epithelium to act as a barrier, it must restrict the free passage of substances through the intercellular spaces. Tight junctions (TJs) act as “gates” that limit permeability [2]. These junctions help regulate paracellular transport via selective permeation of substances into the tissue and acting as a barrier to other external factors from tissue entry. TJs are composed of membrane proteins, such as claudins, occludins, and junctional adhesion molecules, and the cytosolic scaffold zonula occludens (ZO) proteins. Claudins and occludins are connected to the actin cytoskeleton through ZO family proteins [2].

TJ-barrier disruption in nasal and airway epithelial cells has been observed in many pulmonary diseases, such as chronic obstructive pulmonary disease, acute respiratory distress syndrome, lung cancer, asthma, and allergic rhinitis [3]. Viral and bacterial infections, poly (I:C) mimicking viral infections, bacterial endotoxin lipopolysaccharide (LPS) [4], mites, various plant proteases [5], and diesel exhaust particulates, which are a major component of PM2.5 [6], also disrupt the TJ barrier in bronchial and pulmonary epithelial cells. This disruption of the bronchial epithelial-barrier function facilitates the entry of foreign factors into the body, leading to the development or exacerbation of various respiratory diseases [7]. Therefore, maintaining and promoting the epithelial cell barrier function is very important to prevent the onset and exacerbation of various respiratory diseases.

Shoseiryuto (SST) is a Kampo formulation approved by the Ministry of Health, Labor, and Welfare as a treatment for allergic rhinitis, bronchitis, asthma, and cold symptoms [8]. Much evidence has been accumulated regarding the efficacy and mechanism of SST. *In vitro* studies have shown that SST has inhibitory effects on various activities, including histamine release and degranulation of mast cells, eosinophil proliferation, basophil maturation and differentiation, and tumor necrosis factor-*α* (TNF-*α*) synthesis in peripheral blood mononuclear cells [9]. *In vivo* studies have demonstrated that SST is effective against passive cutaneous anaphylaxis (PCA), allergic rhinitis, allergic asthma [9], and anti-influenza or antiviral effects [10]; however, the effect on bronchial TJ-barrier function has not yet been determined.

This study aimed to evaluate the effect of SST on TJ-barrier function. We investigated the effects of SST on TJ-barrier function in human bronchial epithelial cell monolayers cultured in the absence and presence of LPS. It has been reported that transepithelial electrical resistance (TEER) between cell monolayers increases with TJ barrier formation and decreases with weakening. Conversely, intercellular permeability (i.e., paracellular permeability) of small molecules, such as sodium fluorescein (Na-F) and fluorescein isothiocyanate dextran, decreases with barrier formation and increases with barrier weakening [11]. Therefore, we assessed TJ-barrier function by measuring TEER and the Na-F permeability of a cultured-cell monolayer. We also evaluated both the gene mRNA and protein expressions of the TJ protein, occludin.

## 2. Materials and Methods

### 2.1. Test Substance SST

**Figure 1:**
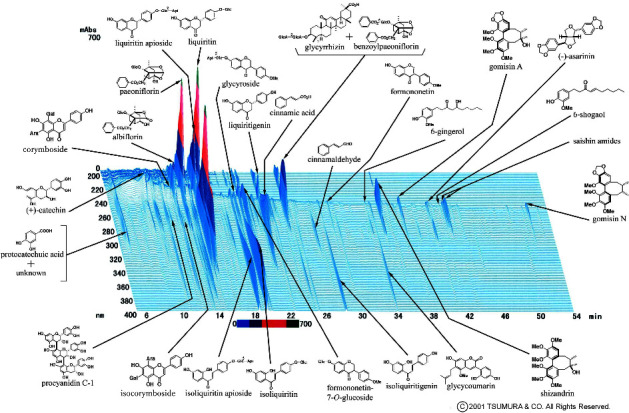
3D-HPLC chromatogram of the SST extract. Pinellia Tuber-derived flavonoids (corymboside and isocorymboside). Processed ginger-derived phenolic compounds (6-shogaol and 6-gingerol). Glycyrrhiza-derived triterpenoid (glycyrrhizin) and flavonoids (liquiritin apioside, liquiritin, liquiritigenin, isoliquiritin apioside, isoliquiritin, isoliquiritigenin, glycyroside, formononetin, formononetin-7-*O*-glucoside, and glycycoumarin). Cinnamon bark-derived phenylpropanoids (cinnamic acid and cinnamic aldehyde) and tannins (procyanidin C-1). Schisandra fruit-derived lignans (schizandrin, gomisin A, and gomisin N) and catechol (protocatechuic acid). Asiasarum root-derived lignin ([−] asarinin) and amides (saishin amides). Peony root-derived monoterpenoids (paeoniflorin, albiflorin, and benzoylpaeoniflorin). Asiasarum root and Ephedra herb-derived tannin (catechin).

The SST used in this study was a dry powdered extract (lot no. 2100019010, Tsumura & Co., Tokyo, Japan) extracted from a mixture of eight crude drugs as follows (Table 1): Pinellia tuber, Processed ginger, Glycyrrhiza, Cinnamon bark, Schisandra fruit, Asiasarum root, Peony root, and Ephedra herb. The quality was standardized according to the Good Manufacturing Practice standards of the Ministry of Health, Labor, and Welfare.  Figure 1 shows a three-dimensional high-performance liquid chromatography chromatogram obtained by ultraviolet detection of the extract, which was provided by Tsumura Co. At least, 28 ingredients were identified.

### 2.2. Materials

#### 2.2.1. Bronchial Epithelial Cell Culture

A human bronchial epithelial cell line 16HBE14o- (16HBE cells) was purchased from Merck KGaA (Saint Louis, MO, USA). A minimum essential medium (MEM) containing 10% fetal bovine serum (FBS) and 1% penicillin-streptomycin mixed solution (PSMS) was used as the culture medium (hereinafter, called as normal medium). MEM, FBS, and PSMS used for the preparation were purchased from Gibco (Grand Island, NY, USA). Trypsin was purchased from Gibco. Culture flasks (75 cm^2^), 24-well transwell plates (Costar®), and collagen-coated 12- and 96-well microplates were purchased from Corning Life Sciences (Acton, MA, USA).

#### 2.2.2. Barrier Assessment

Fluorescein sodium salt (Na–F: MW 376.27) and LPS were purchased from Sigma-Aldrich (Saint Louis, MO, USA). Hanks' balanced salt solution (HBSS) and N-(2-Hydroxyethyl)piperazine-N-2-ethane sulfonic acid (HEPES) buffer were purchased from Gibco.

#### 2.2.3. Immunofluorescence Staining

Phosphate-buffered saline (PBS), bovine serum albumin (BSA), and Tween 20 were purchased from Gibco, Sigma-Aldrich, and Bio-Rad laboratories Inc. (Hercules, CA, USA), respectively. Occludin antibody (F-11, Alexa fluor 594, # SC133255AF594) was purchased from Santa Cruz Biotechnology (Dallas, TX, USA). Prolong Gold antifade reagent with the nuclear staining dye 4′,6-diamidino-2-phenylindole (DAPI) was purchased from Thermo Fisher Scientific (Waltham, MA, USA).

#### 2.2.4. Real-Time Quantitative Polymerase Chain Reaction (RT-qPCR) Analysis

An RNeasy Plus Mini Kit, a ReverTra Ace qPCR RT Kit, and a KOD SYBRR qPCR Mix were purchased from Toyobo Co., Ltd. (Tokyo, Japan).

### 2.3. Experimental Design

#### 2.3.1. Cell Culture

First, 16HBE cells (1 × 10^6^ cells) were seeded into 75 cm^2^ culture flasks with 15 mL of normal medium and incubated at 37°C under 5% CO_2_. After the cells reached full confluency, they were treated with 0.25% trypsin and passaged. Third-passage cells were used for the subsequent experiments.

#### 2.3.2. Determination of the Noncytotoxic Concentrations of SST and LPS on 16HBE Cells

The 16HBE cells (5 × 10^3^ cells/well) were cultured in the normal medium (100 *μ*L/well, control) and medium containing SST (0.03–1.0 mg/mL) or LPS (0.01–3.0 mg/mL) at 37°C under 5% CO_2_ for 4 days (for SST) or 2 days (for LPS) in 96-well microplates. The medium was replaced every day. A cell counting kit-8 (CCK-8) assay was used to measure cell viability to assess the effects of SST and LPS on 16HBE cells on days 4 and 2, respectively.

#### 2.3.3. Barrier-Function Formation in the Bronchial Epithelial Cell Culture

**Figure 2:**
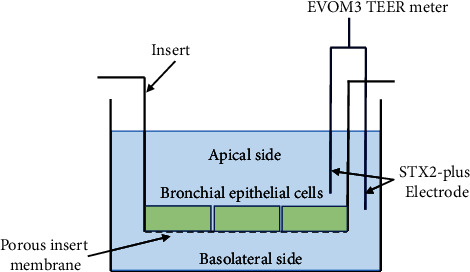
Measurement of TEER in a transwell. A transwell is partitioned into apical and basal compartments by an inserted basket (insert). Bronchial epithelial cells seeded on the upper surface of the porous insert membrane (0.4 *μ*m pore size) were cultured in the culture medium. An EVOM3 epithelial voltage resistance meter with a STX2-plus “chopstick” electrodes was used to measure TEER values.

All barrier formation experiments were performed using a liquid-liquid interface culture system (Figure 2) in a transwell chamber, partitioned into apical and basolateral compartments by an inserted basket (insert). In this experiment, 16HBE cells (1 × 10^5^ cells/well) were seeded onto the upper surface of an insert-well membrane (diameter 6.5 mm, pore size 0.4 *μ*m) in 24-well transwell plates. The cells were cultured at 37°C under 5% CO₂ for 7 days in the normal medium, which was added to both the apical and basolateral compartments. The medium was replaced every day. Cell morphology or confluency was observed daily using a stereomicroscope (ECLIPSE Ts2-FL; Nikon, Tokyo, Japan). TEER values and Na-F permeability were measured daily.

#### 2.3.4. Effect of SST on Epithelial TJ-Barrier Formation and Function

The 16HBE cells (1 × 10^5^ cells/well) were seeded into 24-well transwell plates. The cells were cultured at 37°C under 5% CO₂ for 3 days in the normal medium (control) or medium containing SST (0.03–0.3 mg/mL), which was added to both the apical and basolateral compartments. The medium was replaced every day. TEER values and Na-F permeability measurements and immunofluorescent staining of occludin protein were performed on day 3. In addition, RT-qPCR analysis was performed to assess the occludin mRNA expression on day 3 in a separate set of experiments using 12-well plates (3 × 10^5^ cells/well).

#### 2.3.5. Effects of SST on the LPS-Induced Destruction of the Epithelial TJ Barrier

*(1) Pretreatment Effect of SST*. In this experiment, we set up three groups as follows: control, LPS, and SST + LPS (*n* = 6/each group). In the control group, 16HBE cells (1 × 10^5^ cells/well) seeded in 24-well transwell plates were cultured in the normal medium (both the apical and basolateral compartments) at 37°C and 5% CO_2_ for 5 days. In the LPS group, seeded cells were cultured in the medium for 3 days followed by 2 more days in the medium containing LPS (1 mg/mL). In the SST + LPS group, seeded cells were cultured in the medium containing SST (0.03–0.3 mg/mL) for 3 days followed by 2 more days in the medium containing LPS (1 mg/mL). For the LPS and SST + LPS groups, the medium containing LPS and/or SST was added to the medium in both the apical and basolateral compartments. Each medium was replaced every day. TEER values and Na-F permeability measurements and immunofluorescent staining of occludin protein were performed on day 5. In addition, RT-qPCR was used to assess occludin mRNA expression on day 5 in a separate set of experiments using 12-well plates (3 × 10^5^ cells/well).

*(2) Cotreatment Effect of SST*. In this experiment, three groups (*n* = 6/each group) were set up as follows: control, LPS, and LPS + SST. In the control group, 16HBE cells (1 × 10^5^ cells/well) seeded into 24-well transwell plates were cultured in the normal medium added to both the apical and basolateral compartments for 5 days. In the LPS group, seeded cells were cultured in the same medium for 3 days followed by 2 more days in the medium containing LPS (1 mg/mL). In the LPS + SST group, cells were similarly cultured in the normal medium for 3 days, followed by 2 more days in the medium containing LPS (1 mg/mL) + SST (0.1 and 0.3 mg/mL). For the LPS and LPS + SST groups, the medium containing LPS and/or SST was applied to the apical as well as basolateral compartments. Each medium was replaced every day, and TEER values and Na-F permeability measurements and immunofluorescent staining of occludin protein were performed on day 5. Occludin mRNA expression was assessed by RT-qPCR on day 5 in a separate set of experiments using 12-well plates (3 × 10^5^ cells/well).

*(3) Posttreatment Effect of SST*. In this experiment, three groups (*n* = 6/each group) were also set up as follows: control, LPS, and LPS + SST. As a control group, 16HBE cells (1 × 10^5^ cells/well) seeded into 24-well transwell plates were cultured in the normal medium added to both the apical and basolateral compartments for 9 days. In the LPS group, seeded cells were cultured in the same medium for 3 days, followed by 2 more days from days 3 to 5 in the medium containing LPS (1 mg/mL). Subsequently, the cells were cultured in the LPS-free normal medium for 4 more days from days 5 to 9. In the LPS + SST group, the seeded cells were cultured in the normal medium for 3 days, in an LPS (1 mg/mL)-containing medium for 2 more days, and followed by 4 more days in the medium containing SST (0.1 and 0.3 mg/mL). For the LPS and LPS + SST groups, the medium containing LPS and/or SST was applied to both the apical and basolateral compartments. Each medium was replaced every day, and TEER values and Na-F permeability measurements and immunofluorescent staining of occludin protein were performed on day 9. The occludin mRNA expression was assessed by RT-qPCR on day 9 in a separate set of experiments using 12-well plates (3 × 10^5^ cells/well).

### 2.4. Assay Methods

#### 2.4.1. CCK-8 Assay

The CCK-8 assay was performed according to the manufacturer's protocol for the assay kit (Dojindo Molecular Technologies, Gaithersburg, MD, USA), as previously reported [12]; CCK-8 solution (10 *μ*L/well) was added to wells containing cells. The cells were incubated for 2 h at 37°C in a CO_2_ incubator, and a microplate reader (Thermo Fisher Scientific) was used to measure the absorbance at 450 nm. Cell viability was calculated as a percentage of the absorbance of the SST-treated cells relative to that of the control.

#### 2.4.2. TEER Values and Na-F Paracellular Permeability

TEER values and Na-F permeability were measured according to previously reported procedures [11]. In brief, an EVOM3 epithelial voltage resistance meter (World Precision Instruments, Sarasota, Florida, USA) equipped with the STX2-plus “chopstick” electrodes was used to measure TEER values (Figure 2). In each experiment, after TEER was measured, the apical medium in the insert was replaced with 360 *μ*L of HBSS/HEPES buffer containing 10 *μ*g/mL Na-F, and the basolateral medium was replaced with 1.3 mL of HBSS/HEPES buffer. After incubation at 37°C for 1 h, a fluorometer (Thermo Fisher Scientific) was used to measure the Na-F concentration in the basolateral solution (excitation; 485 nm, emission; 535 nm). The Na-F permeability from the apical side to the basolateral side of the monolayer barrier was calculated as follows:

(1) $$\begin{array}{c} \text{Na}‐F\text{ permeability }\left(\%\right)=\frac{\left(N_{a}-{\text{F}}_{\text{basilareral}}\right)}{\left(N_{a}-{\text{F}}_{\text{apical}}\right)}\times 100, \end{array}$$

where Na‐F_basolateral_ is the absolute amount of Na-F in the basolateral solution after 1 h incubation and Na‐F_apical_ is the absolute amount of Na-F in the apical medium before incubation.

#### 2.4.3. Immunofluorescence Staining

Immunofluorescence staining of the occludin protein was performed according to a previously reported procedure [13]. In brief, after TEER measurement, cells on the transwell insert membrane were fixed in 10% neutral-buffered formalin for 1 h. The cells were then rinsed three times for 10 min each in PBS, 1% BSA, and 0.2% Tween 20 sequentially at room temperature and then incubated with an antioccludin antibody (1 : 100) at 4°C overnight. After washing three times with PBS, the insert membranes attached with cells were mounted onto slides and incubated with Prolong Gold antifade reagent containing DAPI for 1 h. Then, a fluorescence microscope (BZ-X700; Keyence, Osaka, Japan) was used to examine and image the observed immunofluorescence.

#### 2.4.4. RT-qPCR Analysis

RT-qPCR analysis was performed according to a previously reported procedure [12]. An RNeasy plus Mini kit following the manufacturer's instructions was used to isolate the total RNA from the 16HBE cells. ReverTra AceR qPCR RT Kit following the manufacturer's instructions was used to reverse transcribe the RNA (2.0 *μ*g) to cDNA. An Applied BiosystemsR 7500 fast real-time PCR system (Thermo Fisher Scientific K.K., MA, USA) using specific primers and KOD SYBRR qPCR Mix was used to quantify the expression of each gene. The PCR reaction conditions were as follows: predenaturation for 2 min at 98°C, denaturation for 10 sec at 98°C, annealing for 10 sec at 60°C, and extension for 30 sec at 68°C, which was repeated for 40 cycles. The sequences of the forward (F) and reverse (R) primers of each gene were as follows: occludin: (F) 5′-GACACTGGCCTACAGGAATACA-3′ and (R) 5′-ATTCATCAGCAGCAGCCATGT-3′ and GAPDH: (F) 5′-AATCCCATCACCATCTTC-3′ and (R) 5′-AGGCTGTTGTCATACTTC-3′.

The expression level of each gene was expressed relative to that of the control (=1.0) after normalization using glyceraldehyde-3-phosphate dehydrogenase (GAPDH) gene expression.

#### 2.4.5. Statistical Analysis

All data are presented as the mean ± standard error of the mean (SEM). One-way analysis of variance (ANOVA) and post hoc analysis (Dunnett's test) were used to determine the significance of the associations using GraphPad Prism 9 (San Diego, CA, USA). The accepted significance level was *P*  <  0.05.

## 3. Results

### 3.1. Cell Viability Assay to Determine Noncytotoxic Concentrations of SST and LPS

**Figure 3:**
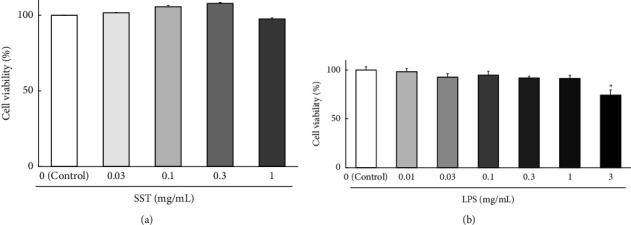
The viability of 16HBE cells cultured for 4 days in a medium containing various concentrations of SST (a) and 2 days in a medium containing LPS (b). The data represent the mean ± SEM (*n* = 6). ^*∗*^*p*  <  0.05 vs. control (one-way ANOVA + Dunnett's test).

Noncytotoxic concentrations were determined by exposing 16HBE cells to varying concentrations of SST (0.03‒1.0 mg/mL) for 4 days (Figure 3(a)) and LPS (0.01‒3.0 mg/mL) for 2 days (Figure 3(b)). No significant difference was observed in the cell viability of each SST group compared with the controls. However, a slightly decreasing trend was observed at the highest concentration of SST (1 mg/mL). LPS significantly decreased cell viability at 3 mg/mL, but no significant changes were observed in a concentration range of 0.01–1.0 mg/mL compared with the control.

### 3.2. Relationship between TEER Values and Na-F Permeability

**Figure 4:**
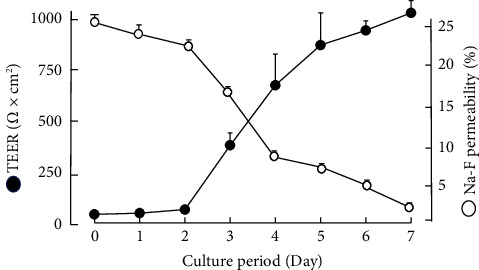
Relationship between TEER values and Na-F permeability over the course of culture days. Each value represents the mean ± SEM (*n* = 6).

Figure 4 shows the results of investigating the process of epithelial cell barrier formation using TEER and Na-F permeability as indices. With the help of stereomicroscopic observation, we confirmed that the cells on the transwell membrane had already reached complete confluence on day 1 after cell seeding. The significant increase in the TEER value and decrease in Na-F permeability were not observed, even at the full confluence state. During the 7-day culture period, TEER values increased with increasing days of culture, and conversely, the Na-F permeability decreased; a clear inverse correlation was observed between them.

### 3.3. Effect of SST on the TJ Function

**Figure 5:**
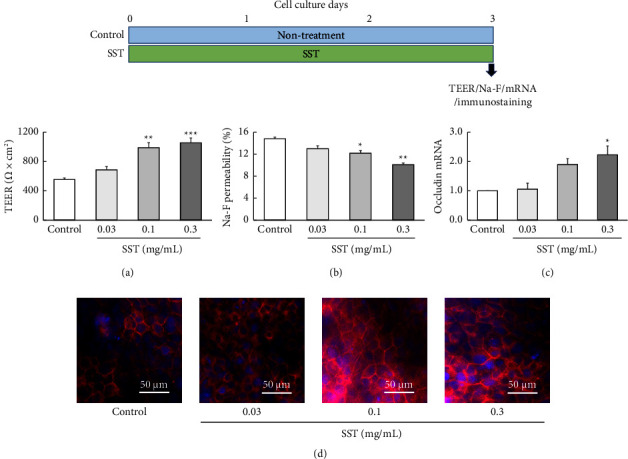
Effect of SST on the TJ function. (a–d) The results of TEER, Na-F permeability, occludin mRNA expression, and immunofluorescent-stained occludin protein on day 3 of culture. Each value represents the mean ± SEM (*n* = 6). ^*∗*^*p*  <  0.05, ^*∗∗*^*p*  <  0.01, and ^*∗∗∗*^*p*  <  0.001 vs. control (one-way ANOVA + Dunnett's test).

To clarify the effect of SST on barrier function formation, cells were cultured in the medium containing SST (0.01–0.3 mg/mL) for 3 days (Figure 5). SST not only significantly increased the TEER value (Figure 5(a)) but also significantly decreased the Na-F permeability (Figure 5(b)) in a dose-dependent manner relative to those in the controls. Moreover, SST significantly increased both the mRNA expression of occludin (Figure 5(c)) and immunofluorescent-stained protein (Figure 5(d)).

### 3.4. Effects of SST on LPS-Induced TJ Disruption

#### 3.4.1. Pretreatment effect of SST

**Figure 6:**
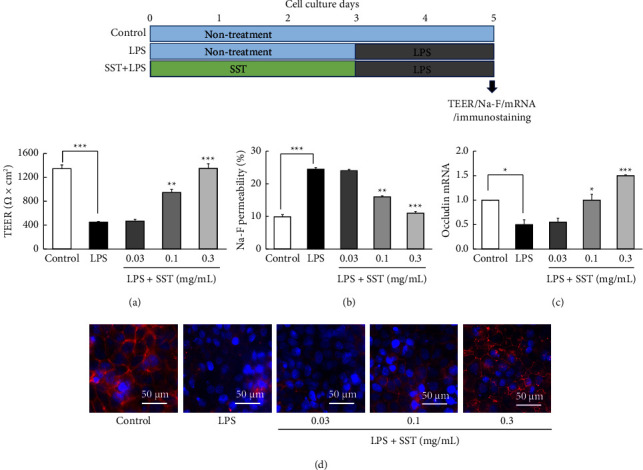
Pretreatment effects of SST on LPS (1 mg/mL)-induced TJ-barrier damage. (a–d) The results of TEER, Na-F permeability, occludin mRNA expression, and immunofluorescent-stained occludin protein on day 5 of culture. Each value represents the mean ± SEM (*n* = 6). ^*∗*^*p*  <  0.05, ^*∗∗*^*p*  <  0.01, and ^*∗∗∗*^*p*  <  0.001 vs. LPS (one-way ANOVA + Dunnett's test).

Figure 6 shows the pretreatment effects of SST on LPS-induced TJ disruption. Two-day LPS (1 mg/mL) exposure from days 3 to 5 significantly decreased the TEER value (Figure 6(a)) and significantly increased Na-F permeability (Figure 6(b)) on day 5 relative to those in each control group. Furthermore, LPS significantly decreased occludin mRNA expression levels (Figure 6(c)). Immunofluorescence staining revealed that the distinct red mesh-like occludin protein between cells was observed in the control group but not in the LPS group on day 5 (Figure 6(d)). However, LPS-treated cells were not destroyed, and their morphology was preserved, including a DAPI-stained blue nucleus. These results indicated that TJs were almost specifically disrupted by LPS exposure without causing cytotoxicity. Pretreatment with SST (0.03–0.3 mg/mL) for 3 days prior to LPS exposure significantly prevented LPS-induced barrier impairment (Figures 6(a)–6(d)) in a dose-dependent manner.

#### 3.4.2. Cotreatment Effect of SST

**Figure 7:**
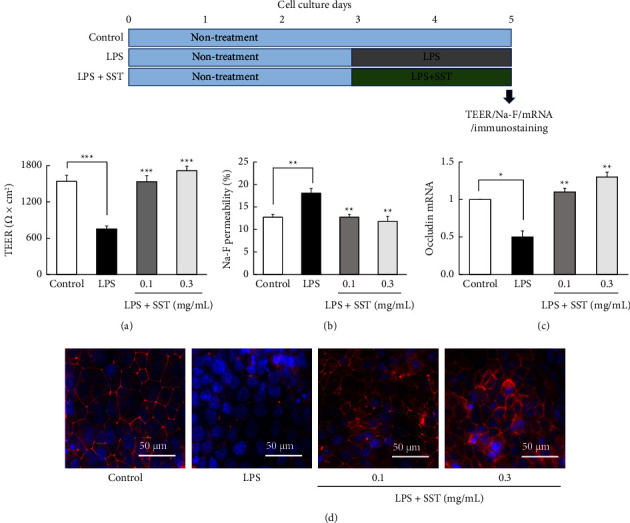
Cotreatment effects of SST on LPS (1 mg/mL)-induced TJ-barrier damage. (a–d) The results of TEER, Na-F permeability, occludin mRNA expression, and immunofluorescent-stained occludin protein on day 5 of culture. Each value represents the mean ± SEM (*n* = 6). ^*∗*^*p*  <  0.05, ^*∗∗*^*p*  <  0.01, and ^*∗∗∗*^*p*  <  0.001 vs. LPS (one-way ANOVA + Dunnett's test).

Figure 7 shows the cotreatment effects of SST on LPS-induced TJ disruption. LPS (1 mg/mL) induced a decrease in TEER values, an increase in Na-F permeability, and a decrease in occludin mRNA as well as protein expression, similar to that observed in subsection 3.4.1. The concomitant use of SST significantly ameliorated these LPS-induced changes, reflecting the TJ-barrier dysfunction (Figures 7(a)–7(d)).

#### 3.4.3. Posttreatment Effect of SST

**Figure 8:**
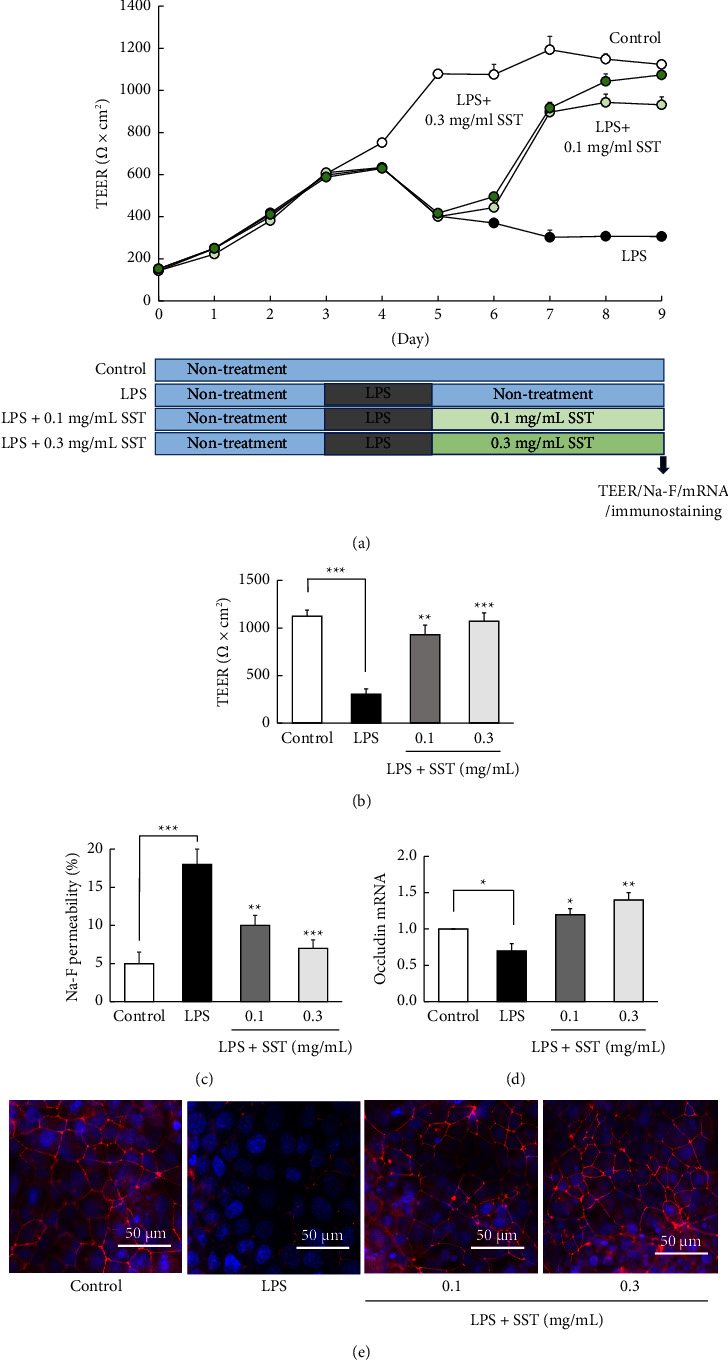
Posttreatment effects of SST on LPS (1 mg/mL)-induced TJ-barrier damage. (a) Culture day-dependent changes in TEER values. (b–e) Results of TEER, Na-F permeability, occludin mRNA expression, and immunofluorescent-stained occludin protein on day 9 of culture. Each value represents the mean ± SEM (*n* = 6). ^*∗*^*p*  <  0.05, ^*∗∗*^*p*  <  0.01, and ^*∗∗∗*^*p*  <  0.001 vs. LPS (one-way ANOVA + Dunnett's test).

Figure 8 shows the posttreatment effects of SST on LPS-induced TJ disruption. The TEER value in the control group increased with increasing culture days, and the increased level remained almost constant from days 5 to 9 (Figure 8(a)). Two days of LPS exposure, from days 3 to 5 of culture, significantly decreased TEER values relative to those in the control group. In the LPS group, the decreased TEER value did not recover even after replacing the LPS medium with the normal medium (days 5–9). In contrast, in the SST group, the TEER values that were decreased by LPS were dose-dependently increased to control levels by replacement with the SST medium (0.1 and 0.3 mg/mL). Figures 8(b)–8(e) show the results of TEER values, Na-F permeability, and occludin mRNA and immunofluorescent-stained protein determined on day 9. Treatment with SST (0.1 and 0.3 mg/mL) alone after LPS exposure significantly ameliorated the LPS-induced decreased TEER values, increased Na-F permeability, and decreased the mRNA expression levels of occludin. Immunofluorescent staining revealed that the distinct red mesh-like occludin protein between cells was observed in the control group but not in the LPS group; however, LPS-treated cells were not destroyed and their morphology was preserved, similar to those observed in Figures 6(d) and 7(d). In contrast, occludin protein was clearly observed when the medium was replaced with SST (0.1 and 0.3 mg/mL) following LPS exposure.

## 4. Discussion

The human 16HBE cells used in this study are a bronchial epithelial cell line, which is widely used as a model for respiratory epithelial diseases and barrier function [14]. Using monolayer 16HBE cells cultured in a transwell liquid-liquid interface culture system (i.e., submerged conditions), we demonstrated that SST promotes the epithelial TJ-barrier formation and has protective and therapeutic effects against LPS-induced TJ-barrier damage. Recently, an air-liquid interface (ALI) culture system has suggestively been useful for studying the respiratory system and is thought to be suitable for studying airway epithelial cells that interact with both liquid and air because one side of the culture surface is in contact with the medium, whereas the other side is in contact with air [15]. In addition, the 16HBE cells cultured under ALI conditions exhibit biological polarity, such as apical expression of chloride channels as well as mucosal secretory and ciliated cells [15]. However, it has been reported that 16HBE cells cultured under ALI conditions show insufficient TJ protein formation and the TEER value is 69% lower compared with that of cells cultured under submerged conditions [16]. In this study, we aimed to clarify the effects of SST on TJ formation. Therefore, we selected a liquid-liquid interface culture system, which allows for the formation of stable and robust TJs. Therefore, our findings are based on the experimental conditions used in this study.

In the cell-culture experiments, noncytotoxic concentrations must be used to accurately assess the efficacy of test substances [11, 12]. The maximum cell-exposure duration for SST in this experimental design was 4 days. Therefore, noncytotoxic concentrations were determined by exposing 16HBE cells to varying concentrations of SST (0.03‒1.0 mg/mL) for 4 days (Figure 3). SST concentrations in this range are nontoxic to 16HBE cells. However, a slightly decreasing trend was observed at the highest concentration of SST (1 mg/mL). Therefore, in subsequent experiments, we used 0.03–0.3 mg/mL concentrations of SST. The concentration of LPS (1 mg/mL) used to induce TJ-barrier dysfunction in the present study was high compared to those used in other studies. Previously, we used LPS at a concentration of 100 *μ*g/mL to induce disruption of the Caco-2 cell intestinal epithelial barrier [11] and blood-brain barrier formed by the coculture of rat brain endothelial cells, pericytes, and astrocytes [17]. This may be attributed to the differences in cell types and experimental conditions. Nonetheless, it is important to clarify whether or not the LPS concentration required for induction of TJ-barrier dysfunction is cytotoxic. As shown in Figures 6–8, TJ-barrier dysfunction was induced by exposure to LPS at a concentration of 1.0 mg/mL for 2 days. Therefore, we determined the effects of LPS on cells (Figure 3(b)) and confirmed that cytotoxicity was not induced by exposure to 1.0 mg/mL LPS for 2 days. In preliminary studies, LPS concentrations below 1.0 mg/mL did not induce barrier dysfunction (data not shown here). Therefore, the optimal concentration of LPS was selected to be 1.0 mg/mL of LPS for specifically inducing TJ-barrier dysfunction without causing cytotoxicity.

When the formation process of the epithelial cell barrier was investigated using TEER and Na-F permeability as indices (Figure 4), the TEER value increased with increasing days of culture, while the Na-F permeability decreased. This inverse correlation suggests that the TJ barrier is reliably formed as the number of culture days increases [11] under these experimental conditions. In this experiment, we confirmed that the cells seeded into the transwell reached full confluence on day 1; however, an increased TEER value and decreased Na-F permeability were not observed, even in the fully confluent state. This suggests that continuous culture after the cells reaching full confluence is necessary for TJ-barrier formation.

Figures 4 and 8 show that the increase in TEER values reached a plateau after day 5 of culture, suggesting that TJ-barrier formation was complete. Therefore, it was thought to be difficult to further assess the promoting effects of SST after the barrier was already completed. In contrast, the midpoint of barrier completion, i.e., day 3 or 4 of culture, would be an optimal time for determining whether SST inhibits or promotes the TJ-barrier formation.

Therefore, to clarify the effect of SST on barrier-function formation, cells were cultured in a medium containing SST for 3 days (Figure 5). SST significantly increased the TEER value, decreased Na-F permeability, and increased both the mRNA and protein expression levels of occludin. These results suggest that SST promotes TJ-barrier formation likely by promoting protein expression following the upregulation of occludin mRNA (a direct promoting action of TJ formation).

Various pulmonary diseases as well as viral and bacterial infections induce oxidative stress and immunological/inflammatory responses [18], which disrupt the epithelial TJ-barrier in the nose and airways [3, 4]. Destruction of the barrier facilitates entry of foreign substances into the body, leading to the onset and exacerbation of various respiratory diseases [7, 18]. Therefore, promoting the epithelial barrier formation is very important to prevent the onset and exacerbation of various respiratory diseases.

Therefore, we next determined whether SST prevents TJ disruption induced by LPS. LPS exposure significantly decreased the TEER value, increased Na-F permeability, and decreased both occludin mRNA and protein expression (Figures 6 and 7). These results suggest that TJs were destroyed by LPS exposure. Pretreatment (Figure 6) and cotreatment (Figure 7) with SST significantly protected changes in all parameters suggestive of LPS-induced TJ destruction. These results suggest that SST has a protective effect against LPS-induced TJ disruption either during pretreatment or during cotreatment.

Endotoxin LPS, a component of the outer wall of Gram-negative bacteria, promotes the production of excessive amounts of reactive oxygen species and inflammatory, such as TNF-*α*, interferon-*γ*, and interleukins, and subsequently causes barrier dysfunction [19]. Considering such responses of LPS, the protective effect of SST on LPS-induced TJ-barrier disruption may occur via antioxidant and anti-inflammatory mechanisms in addition to previously described direct barrier-promoting effects. Plants and crude drugs contain many bioactive substances, such as phenolic and polyphenolic compounds, which have antioxidant and anti-inflammatory effects [20]. SST consists of eight crude drugs (Table 1) and many active components, including phenols, polyphenols (such as flavonoids and tannins), terpenoids, and phenylpropanoids or their polymer lignans (Figure 1). We speculate that these components are candidates for antioxidant/anti-inflammatory active components responsible for the TJ protective action of SST.

Finally, we determined if SST has a therapeutic effect on LPS-induced TJ disruption (Figure 8). LPS (1 mg/mL) exposure significantly decreased TEER values, which did not recover even after replacing the LPS medium with a normal medium. As mentioned above, 1 mg/mL LPS was a noncytotoxic concentration to the cells (Figure 3(b)). Immunofluorescence revealed that cells exposed to this LPS concentration were not destroyed and their morphology was preserved (Figures 6–8). This suggests that the cells exposed to this concentration remained viable and that the failure of barrier function to recover, even after replacement with normal medium post-LPS exposure, was not due to LPS-induced cytotoxicity (Figure 8). Considering that LPS impaired occludin mRNA and protein expression, LPS likely induced TJ-barrier dysfunction by down-regulating occludin mRNA and subsequently inhibiting protein expression. Although the detailed mechanism is unknown, this suggests that LPS-induced TJ-barrier dysfunction persists for some time after induction. Moreover, if cells are killed by LPS, replacement with the SST-supplemented medium should not restore barrier function. However, treatment with SST after LPS exposure significantly ameliorated the LPS-induced decreased TEER values. This result supports the survival of cells exposed to LPS as well as therapeutic effect of SST on LPS-induced TJ disruption. This therapeutic effect using TEER as an indicator was also supported by the results that SST ameliorated the LPS-induced increase in Na-F permeability and decrease in occludin mRNA and protein expression levels. This therapeutic effect probably involves the direct promoting effect of TJ formation rather than antioxidant/anti-inflammatory action, similar to the results shown in Figure 5. This mechanism can be explained if a TJ barrier that is destroyed by LPS is reformed by SST alone.

Conversely, one might speculate that this therapeutic effect of SST (Figure 8) may be the result of cell proliferation. However, we have already verified that SST has no proliferative effect (Figure 3(a)). Furthermore, as mentioned above, no intercellular occludin protein was observed in the cell layer after exposure with LPS; however, the cells were not destroyed and the cell layer remained fully confluent. Overall, these results suggest it to be unlikely that SST promotes cell proliferation; therefore, proliferation is unlikely to be responsible for the therapeutic effect of SST.

To our knowledge, no active crude drugs or ingredients responsible for this direct therapeutic effect of SST have been reported. However, oral SST has been reported to inhibit PCA responses, and Asiasarum root, Pinellia Tuber, and Glycyrrhiza among the eight crude drugs comprising SST contributed to the antiallergic effect [21]. In addition, Ephedra extract, cinnamaldehyde in Cinnamon bark, and gingerol in Processed ginger, which are components of SST, have been reported to exhibit antiallergic effects [8]. These active crude drugs and ingredients, as well as the antioxidant and anti-inflammatory active ingredients mentioned above, may be candidates for the active crude drugs or ingredients responsible for this direct therapeutic effect of SST.

This study had some limitations. First, we only studied changes in occludin for TJs. Further analyses are required to determine if SST is associated with other TJ-associated components, such as claudin, ZO proteins, and an intercellular adhesion molecule, E-cadherin. Second, this study focused on the effects of SST. Unfortunately, we could not yet verify the constituent crude drugs and ingredients using the barrier dysfunction model. Further studies are required to identify the active ingredients. Finally, the *in vitro* design of the study has inherent limitations. These experiments are difficult to faithfully reproduce the complex cellular composition and physiological state of the tracheal epithelial cell layer *in vivo*. Furthermore, considering that SST is an oral drug, the pharmacokinetics that affects its efficacy in bronchial epithelial cells cannot be ignored. Thus, future studies are required to validate that *in vitro* results are reflected *in vivo* using respiratory disease models, such as lung-injury animal models [22].

## 5. Conclusion

SST promoted TJ-barrier function in 16HBE cells by enhancing occludin mRNA levels and protein expression. Moreover, SST protected and restored LPS-induced TJ-barrier damage. These protective and therapeutic effects conferred by TJ-barrier promoting, a novel mechanism supporting the efficacy of SST for respiratory diseases, have not been previously reported.

## Figures and Tables

**Table 1:** Composition of shoseiryuto

Crude drugs	Scientific names	Content (% ratio)
Pinellia tuber	*Pinellia ternate* Breitenbach	6.0 g (22.3%)
Processed ginger	*Zingiber officinale* Roscoe	3.0 g (11.1%)
Glycyrrhiza	*Glycyrrhiza uralensis* Fischer	3.0 g (11.1%)
Cinnamon bark	*Cinnamomum cassia* J. Presl	3.0 g (11.1%)
Schisandra fruit	*Schisandra Chinensis* Baillon	3.0 g (11.1%)
Asiasarum root	*Asiasarum heterotropoides* F. Maekawa var. *mandshuricum* F. Maekawa	3.0 g (11.1%)
Peony root	*Paeonia lactiflora* Pallas	3.0 g (11.1%)
Ephedra herb	*Ephedra sinica* Stapf	3.0 g (11.1%)

## Data Availability

The data used to support the findings of this study are available from the corresponding author on reasonable request.

## Abbreviations

ALI: Air-liquid interface
BSA: Bovine serum albumin
DAPI: 4′,6-diamidino-2-phenylindole
FBS: Fetal bovine serum
GAPDH: Glyceraldehyde-3-phosphate dehydrogenase
HBSS: Hanks' balanced salt solution
HEPES: N-(2-hydroxyethyl) piperazine-N-2-ethane sulfonic acid
LPS: Lipopolysaccharide
MEM: Minimum essential medium
Na-F: Sodium fluorescein
PBS: Phosphate-buffered saline
PCA: Passive cutaneous anaphylaxis
PSMS: Penicillin–streptomycin mixed solution
SST: Shoseiryuto
TEER: Transepithelial electrical resistance
TJ: Tight-junction
TNF-*α*: Tumor necrosis factor-*α*
ZO: Zonula occludens.
